# Food insecurity and its associated factors among lactating mothers in the Chiro district, Eastern Ethiopia: A community-based cross-sectional study

**DOI:** 10.3389/fnut.2022.922774

**Published:** 2022-10-04

**Authors:** Selamu Minas, Behailu Hawulte Ayele, Mekonnen Sisay, Biruk Shalmeno Tusa, Kedir Teji Roba

**Affiliations:** ^1^West Hararghe Zonal Health Department, Chiro, Ethiopia; ^2^School of Public Health, College of Health and Medical Sciences, Haramaya University, Harar, Ethiopia; ^3^School of Pharmacy, College of Health and Medical Sciences, Haramaya University, Harar, Ethiopia; ^4^School of Nursing and Midwifery, College of Health and Medical Sciences, Haramaya University, Harar, Ethiopia

**Keywords:** food insecurity, associated factors, lactating mothers, Chiro, Ethiopia

## Abstract

**Introduction:**

Lactating mothers are extremely vulnerable to both macro and micronutrient deficiencies due to the increased nutritional requirements and high magnitude of food insecurity in low-income countries. However, there are a dearth of studies conducted in sub-Saharan African countries regarding this study area. Thus, this study aimed to assess the magnitude of food insecurity and its associated factors among lactating mothers in the Chiro district, eastern Ethiopia.

**Methods:**

A community-based cross-sectional study was conducted among 446 randomly selected lactating mothers from 1–30 June, 2020. Data were collected through face-to-face interviews using a structured and pre-tested questionnaire. Data were entered using EpiData version 3.1 and exported to STATA version 14.2 for cleaning and analysis. Bi-variable and multivariable binary logistic regression analyses were fitted to check the association between independent variables and food insecurity. The level of statistical significance was declared at a *p*-value < 0.05.

**Results:**

The magnitude of food insecurity among lactating mothers was 68.8 % (95 % CI: 64.4, 72.9) and 12.1% (95 % CI: 9.4, 15.5) were severely food insecure. Residing in the rural (AOR =2.36, 95% CI:1.21, 4.62), poor wealth indices (AOR =4.68, 95% CI:2.02, 10.8), owning farmland of less than a hectare (AOR =2.35, 95% CI:1.06, 5.19), mothers who had less than three meals a day (AOR =2.70, 95% CI:1.33, 5.46), and who did not have their own income (AOR =2.32, 95% CI:1.36, 3.96) were significantly associated factors with food insecurity among lactating mothers.

**Conclusion:**

Food insecurity is highly prevalent in lactating mothers' households. Therefore, the government and other stakeholders need to take action that addresses factors affecting mothers' food security status through strengthening nutrition-sensitive interventions.

## Introduction

Food security is a situation that exists when all people, at all times, have physical, social, and economic access to sufficient, safe, and nutritious food that meets their dietary needs and food preferences for an active and healthy life. The four key dimensions of food security are availability of food, economic and physical access to food, adequate food utilization, and having sustained access to adequate food. Food insecurity exists when one or more of the food security key dimensions fail to be fulfilled (1).

Food insecurity remains highly prevalent in developing countries and has consistently increased. Since 2014, it has been recognized as a serious public health problem in both developing and developed countries. Based on a Food and Agriculture Organization report, globally, an estimated two billion people suffered from food insecurity in 2019. Out of these, 1.03 billion are in Asia, 675 million in Africa, 205 million in Latin America and the Caribbean, 88 million in North America and Europe, and 5.9 million in Oceania (2).

In Ethiopia, food insecurity is a major public health problem, with approximately 26 million people experiencing it, and more than 10.2 and 7.9 million people targeted with life-saving food assistance and the Productive Safety Net Program, respectively (3). Further, the existing highest poverty is considered a major challenge in achieving the sustainable development goal aims to eradicate hunger and all types of malnutrition by 2030 (4).

Lactating mothers need more nutritious food because lactation puts high demands on maternal stores of energy and protein than in any other stage of a woman's reproductive life in order to satisfy their own physical needs and those of their child. Despite the increased nutritional requirements, lactating mothers are extremely vulnerable to both macronutrient and micronutrient deficiencies in Africa (5, 6). Food insecurity affects the intake of adequate quantity and quality of diet that in turn contributes to maternal undernutrition (7).

At the global level, and more markedly in Africa, the magnitude of food insecurity is higher among women than men. Furthermore, the gender gap in food insecurity is higher among those women in the lowest income quintile, with lower education, who are unemployed, with health problems, living in rural areas, or are separated or divorced (2). These findings point to the need for a deeper understanding of the factors that make access to food more difficult for women, even when they live in similar areas as men.

In Ethiopia, despite the government's effort to implement the Productive Safety Net Program to reduce food insecurity at the household level, the high rates of food insecurity persist. In the region, the magnitude of food insecurity ranges from 27 to 71% (8, 9). However, most of these studies were conducted among household heads, particularly male subjects, and there is a dearth of studies among lactating mothers in Ethiopia, specifically in the current study area. Therefore, this study aimed to assess the magnitude of food insecurity and its associated factors among lactating mothers in the Chiro district, Eastern Ethiopia.

## Methods

### Study setting

The study was conducted in the Chiro district, West Hararghe Zone, Eastern Ethiopia. The district is located 326 km from Addis Ababa, the capital of Ethiopia. The district has 39 rural and three urban kebeles (the lowest administrative units in Ethiopia) with an estimated population of 236,091 and 57,873, respectively. Khat is the main economic cash crop widely produced with sorghum and maize mixed farms.

### Study design and population

A community-based cross-sectional study was carried out from June 1 to 30, 2020 among lactating mothers who had children between 6 and 23 months of age.

### Sample size and sampling procedure

A sample size of 497 was obtained using the single population proportion formula, assuming a 26.7% proportion of food insecurity among lactating mothers based on the study conducted in Dire Dawa, Ethiopia (8), 0.05 margin of error, 95% confidence interval, 10%, non-response rate, and design effect of 1.5.

A multistage sampling technique was employed to select the final study units. In the first stage, one out of three urban kebeles and five out of 39 rural kebeles were selected randomly using a lottery method. Second, households with lactating mothers were identified and registered separately in each selected kebele through house-to-house visits by health extension workers. Then, the total sample size was distributed to the kebeles proportionally. Finally, a simple random sampling technique was used to select the required number of lactating mothers using registration as a sampling frame. If eligible lactating mothers were not available at the time of data collection, a revisit was made a minimum of three times, and these women were finally considered non-respondents.

### Data collection tool and procedures

Data were collected using a structured questionnaire adapted from applicable works of literature. Household food insecurity information was collected using the questionnaire adopted from the Household Food Insecurity Access Scale (HFIAS) measurement tool, which is developed by Food and Nutrition Technical Assistant Project (FANTA). The tool consists of nine questions that show the frequency of occurrence and measure the severity of food insecurity in the last 4 weeks in terms of Likert scale question responses (0=never, 1=rarely, 2=sometimes, and 3=often). Then the households were categorized into four levels of household food insecurity (access): food secure, mild, moderately, and severely food insecure (1). The household wealth index was computed using principal component analysis (PCA) as a composite indicator of household living standards based on the ownership of fixed assets. Finally, wealth tercile was performed and categorized as higher, medium, and lower (10). The questionnaire was first prepared in English and then translated into the local language - Afaan Oromoo, and then translated back to English by a language expert to check the consistency. The data were collected by two diploma nurses and six health extension workers who were fluent in the local language through face-to-face interviews at the respondents' homes.

### Operational definitions

#### Data quality assurance

The study tool was pre-tested on 5% of the estimated sample size in a similar setting outside the study area, and necessary corrections were made before the actual collection of the data. The data collectors and supervisors were trained for 2 days on the objectives of the study, interview techniques, ethical issues, and data collection tools. The actual data collection was closely supervised by the principal investigator, and the supervisors checked for completeness and consistency on a daily basis. Double data entry was done by two data clerks who were blind to each other (Table 1).

**Table 1:** Operational definition of the lactating mother in Chiro district, Eastern Ethiopia June 1–30, 2020 (*n* = 446).

**Lactating mother:**	Mothers aged 15–49 years who have children 6–23 months of age, and currently expressing breast milk for their infant/child (11).
**Food secure mothers:**	Mothers who have experienced none of the food–insecure (access) conditions or have just been worried, although rarely, during the past 4 weeks (1).
**Food insecure mothers:**	Mothers who are unable at all times to access food sufficient to lead an active and healthy life (includes all stages of food insecurity; mild, moderate, and severe) (1). Food insecurity was coded as 1 if households of the lactating mother are food insecure and coded as 0 if food secure.
**Mildly food–insecure mothers:**	Mothers who worry about not having enough food sometimes or often and/or are unable to eat preferred foods and/or eat a more monotonous diet than desired and/ or some foods considered undesirable, but only rarely (1).
**Moderately food-insecure mothers:**	Mothers who sacrifice quality more frequently, by eating a monotonous diet or undesirable foods sometimes or often, and/or have started to cut back on quantity by reducing the size of meals or number of meals, rarely or sometimes. However, they do not experience any of the three most severe conditions (1).
**Severely food-insecure mothers:**	Mothers who have been forced to cut back on the meal size or meals often and/or experience any of the three most severe conditions (running out of food, going to bed hungry, or going a whole day and night without eating), even as infrequently as rarely (1).

#### Data processing and statistical analysis

The collected data were checked for completeness and consistency, and entered into EpiData version 3.1, and exported to STATA version 16,0 for cleaning and analysis. Descriptive statistics were used to describe the outcome and explanatory variables. A binary logistic regression model was used to examine the association between the dependent variable (food insecurity) and each independent variable. Accordingly, covariates with a p-value of < 0.25 in the bi-variable binary logistic regression were considered candidates for a multivariable binary logistic regression to control for potential confounders and to identify factors independently associated with household food insecurity. Odds ratio (OR) along with a 95% confidence interval (CI) was estimated to measure the strength of the association. The level of statistical significance was declared at a *p*-value < 0.05. The fitness of the model was tested by the Hosmer–Lemeshow goodness of fit test at a *p*-value < 0.05. Finally, the findings were presented using frequencies, summary measures, tables, and figures.

## Results

### Socio-demographic characteristics of lactating mothers

A total of 446 lactating mothers participated in this study with a response rate of 90%. Half (50.4%) of the mothers were in the age group of 26–35 years, and the mean age was 28.3 (SD ± 6.0) years. Two-thirds of the mothers (67.5%) and 49.9% of the partners had never attended any formal education. About 58.7% of the respondents had more than four family members. Around one-third, 33.6 and 33.4%, of the households were in the poor and medium family wealth index, respectively (Table 2).

**Table 2:** Socio-demographic characteristics of the lactating mother in Chiro district, Eastern Ethiopia June 1–30, 2020 (*n* = 446).

**Variables**	**Categories**	**Frequency (*n* = 446)**	**Percent (%)**
Age of mother in years	15–26	161	36.1
	26–36	255	50.4
	36–50	60	13.5
Place of residence	Urban	128	28.7
	Rural	318	71.3
Religion of mother	Christian	99	22.2
	Muslim	347	77.8
Marital status	Currently married	421	94.4
	Currently unmarried	25	5.6
Mother educational status	No formal education	301	67.5
	Formal education	145	32.5
Partner educational status	No formal education	210	49.9
	Formal education	211	50.1
Mother occupation	Farmer	103	23.1
	Housewife	175	39.2
	Merchant	119	26.7
	Government employee	40	9
	Others^*^	9	2
Partner occupation	Farmer	269	63.9
	Merchant	55	13.1
	Daily laborer	32	7.6
	Government employee	58	13.8
	Others^**^	7	1.7
Family size	≤ 4	184	41.3
	5 and above	262	58.7
Have own source of income^***^	Yes	168	37.7
	No	278	62.3
Farmland ownership (hectares)	< 1 hectares	405	90.8
	≥1 hectares	41	9.2
Family wealth index	Rich	147	33
	Medium	149	33.4
	Poor	150	33.6

* Daily laborer, Private work;

** Driver, guard;

*** Employed or merchant.

### Feeding practice and healthcare service utilization of mothers

Out of the total lactating mothers, 55.8% of them had information about feeding practices during lactation. In addition, more than two-thirds (67.5%) of the mothers had three meals per day. The mean age of the lactating mothers at first pregnancy was 18.0 (SD ± 2.9) years. About 77.2% of the lactating mothers had at least one antenatal care (ANC) visit during their last pregnancy, but only 11.2% of the mothers got four visits to ANC. Moreover, 52.2% of the mothers delivered in a health center (Table 3).

**Table 3:** Feeding practice and healthcare service utilization of lactating mother in Chiro district, Eastern Ethiopia, June 1–30, 2020 (*n* = 446).

**Variables**	**Categories**	**Frequency (*n* = 446)**	**Percent (%)**
Have nutrition information /education	Yes	249	55.8
	No	197	44.2
Frequency of meals per day	Less than three meals	145	32.5
	Three or more meals	301	67.5
Khat Chew	Yes	223	50.0
	No	223	50.0
Age at first pregnancy	< 18 years	228	51.1
	≥18 years	218	48.9
Number of ANC visits	No visit	101	22.7
	< 4 visit	295	66.1
	≥4 visit	50	11.2
Place of delivery	Home	213	47.8
	Health facility	233	52.2
Latrine availability	Yes	329	73.8
	No	117	26.2

### The magnitude of household food insecurity

**Figure 1:**
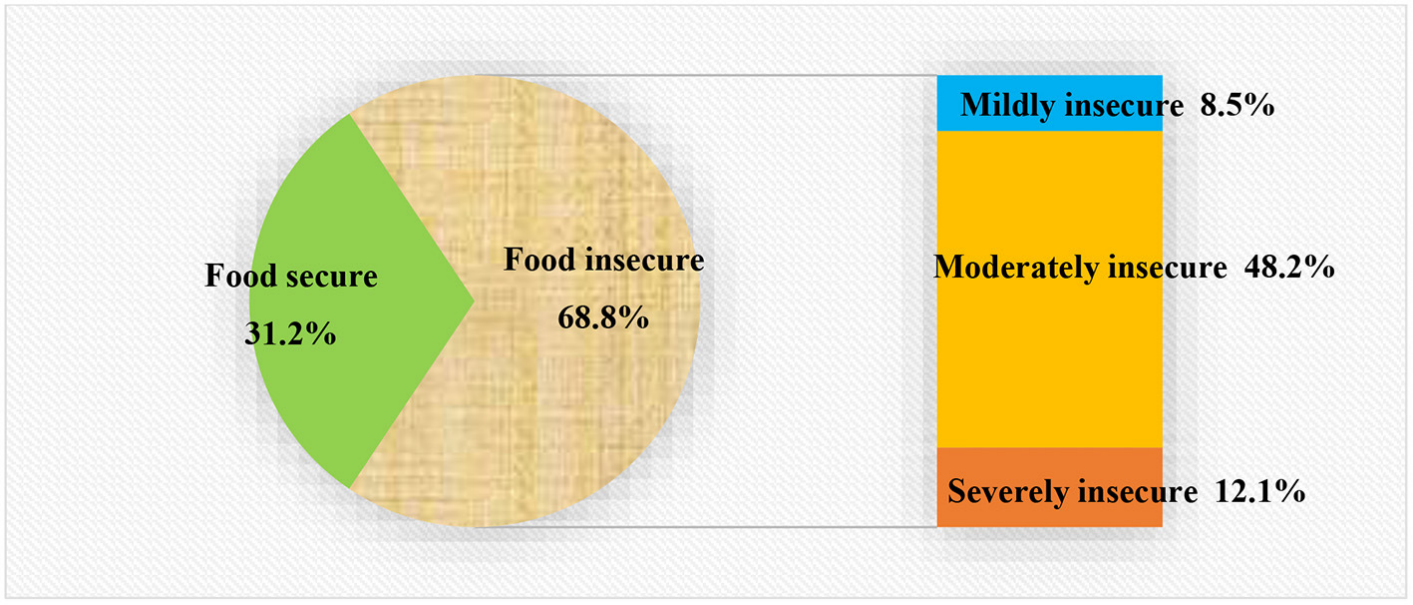
Household food security status of the lactating mother in Chiro district, Eastern Ethiopia, 2020.

Based on set cutoff points, the magnitude of food insecurity among lactating mothers in the Chiro district was 68.8 % (95 % CI: 64.4, 72.9) and 12.1% (95 % CI: 9.4, 15.5) were severely food insecure. The level of household food insecurity was further categorized based on its severity (Figure 1).

### Associated factors of food insecurity among lactating mothers

Based on the binary logistic regression analysis, place of residence, household wealth index, owning farmland, meal frequency, and having own source of income were significantly associated with food insecurity among lactating mothers (Table 4).

**Table 4:** Covariates of food insecurity among lactating mothers in Chiro district, Eastern Ethiopia, June 1–30, 2020 (*n* = 446).

**Independent variables**	**Food security status**		**COR (95%CI)**	**AOR (95%CI)**
	**Insecure (%)**	**Secure (%)**		
Age of mother in years				
15–25	97 (60.2%)	64 (39.8%)	1	1
26–35	162 (72.0%)	63 (28.0%)	1.70 (1.10, 2.61)^*^	1.22 (0.63, 2.33)
36–49	48 (80.0%)	12 (20.0%)	2.64 (1.30, 5.35)^*^	1.27 (0.48, 3.39)
Place of residence				
Urban	48 (37.5)	80 (62.5)	1	1
Rural	259 (81.4)	59 (18.6)	7.32 (4.64, 11.54)^*^	2.36 (1.21, 4.62)^*^
Mother educational status				
Formal education	69 (47.6)	76 (52.4)	1	1
No formal education	238 (79.1)	63 (20.9)	4.16 (2.71, 6.38)^*^	1.66 (0.93, 2.99)
Family size				
≤ 4	102 (55.4)	82 (44.6)	1	1
5 and above	205 (78.2)	57 (21.8)	2.90 (1.91, 4.37)^*^	1.02 (0.49, 2.11)
Family wealth index				
Rich	61 (41.5)	86 (58.5)	1	1
Poor	138 (92.0)	12 (8.0)	16.2 (8.26, 31.8)^*^	4.68 (2.02, 10.8)^*^
Medium	108 (72.5)	41 (27.5)	3.71 (2.28, 6.04)^*^	1.41 (0.74, 2.69)
Age at first pregnancy				
≥18 years	127 (58.3)	91 (41.7)	1	1
< 18 years	180 (79.0)	48 (21.0)	2.68 (1.77, 4.07)^*^	0.83 (0.43, 1.57)
Place of delivery				
Health facility	133 (57.1)	100 (42.9)	1	1
Home	174 (81.7)	39 (18.3)	3.35 (2.17, 5.17)^*^	1.07 (0.60, 1.93)
Latrine availability				
Yes	209 (63.5)	120 (36.5)	1	1
No	98 (83.8)	19 (16.2)	2.96 (1.72, 5.08)^*^	0.83 (0.42, 1.64)
Have nutrition information				
Yes	154 (61.8)	95 (38.2)	1	1
No	153 (77.7)	44 (22.3)	2.15 (1.41, 3.27)^*^	1.27 (0.75, 2.15)
Frequency of meals per day				
Three or more meals	176 (58.5)	125 (41.5)	1	1
Less than three meals	131 (90.3)	14 (9.7)	6.65 (3.65, 12.10)^*^	2.70 (1.33, 5.46)^*^
Mother khat chew				
No	137 (61.4)	86 (38.6)	1	1
Yes	170 (76.2)	53 (23.8)	2.01 (1.34, 3.03)^*^	1.59 (0.96, 2.63)
Having own source of income				
Yes	97 (57.7)	71 (42.3)	1	1
No	210 (75.5)	68 (24.5)	2.26 (1.50, 3.41)^*^	2.32 (1.36, 3.96)^*^
Farmland owned (hectares)				
≥1 hectors	21 (51.2)	20 (48.8)	1	1
< 1 hectors	286 (70.6)	119 (29.4)	2.29 (1.20, 4.38)^*^	2.35 (1.06, 5.19)^*^

* P-value < 0.05; 1, reference of the variable; CI, confidence interval; COR, crude odds ratio; AOR, adjusted odds ratio.

Explicitly explained, mothers residing in rural areas were two times more likely to experience food insecurity than their counterparts in urban areas (AOR =2.36, 95% CI: 1.21, 4.62). Although those mothers who had less than three meals per day were almost three times more likely to be food insecure than those who had three meals and above (AOR =2.70, 95% CI:1.33, 5.46). Moreover, lactating mothers from households who had poor wealth indices were more than four times more likely to experience food insecurity than mothers from rich households (AOR =4.68, 95% CI:2.02, 10.8). Farmland size was also associated with food insecurity; households owning less than a hectare had an increased risk of being food insecure by two times when compared to households owning at least one hectare (AOR =2.35, 95% CI:1.06, 5.19). The odds of becoming food insecure were two times higher among mothers who did not have their source income than mothers who had their source of income (AOR =2.32, 95% CI: 1.36, 3.96).

## Discussion

This study assessed the magnitude of food insecurity and associated factors in the study area among lactating mothers using HFIAS. Accordingly, the magnitude of food insecurity among lactating mothers was 68.8%; and residing in rural areas, having their own source of income, meal frequency of less than three times per day, households with poor wealth indices, and having farmland of less than a hector were significant predictors of household food insecurity.

The magnitude of household food insecurity reported in this study was comparable with the study done in Lay Gayint District, South Gondar zone, Amhara region (70%), and rural Tigray (71%) (9, 12). But, it was lower than a study conducted in rural Malawi, which reported an 86.1% magnitude of food insecurity among lactating mothers (13). This might be due to differences in the study areas and seasonal variation because food security varies from season to season.

On the contrary, the finding observed in this study was much higher than the findings of the studies conducted in Bajhang District in Nepal (54%), and in Moyale district (46.6%), Ataye district (36.8%), Ambo district (38.4%), and Dire Dawa (26.7%) in Ethiopia (5, 7, 8, 14, 15). The disparity might be due to the agroecological differences and socioeconomic variations among study areas. Another possible explanation for the difference could be the effect of desert locust outbreaks and the COVID-19 pandemic, where food is costly and not readily available.

According to our study, mothers residing in rural areas were more likely to experience food insecurity than mothers in urban areas. This is in line with the study conducted in the East and West Gojjam zones of the Amhara Region (16). This is because mothers residing in urban areas might have constant monthly income and also engage in income-generating activities, which increases their purchasing capacity and access to food.

The study also shows that mothers with a poor wealth index were at greater risk of being food insecure than mothers with a rich wealth index. This could be because poor households, accompanied by low income, are not able to purchase adequate foods that can satisfy the required needs of their household members. This study is in agreement with the study conducted in Areka Town, Southern Ethiopia (17).

Similarly, lactating mothers who have no own source of income were two times more likely to be food insecure when compared to mothers who have an income. That was comparable with findings from Ataye District in Ethiopia, and Nepal (5, 14). This can be explained by the fact that the engagement of mothers is endowed with additional income which augments the household economy that can, in turn, increase the purchasing power.

Although, the size of farmland was significantly associated with household food insecurity; households owning less than a hectare had an increased risk of being food insecure when compared to households owning at least one hectare. This might be because households with larger farmland could harvest more food, and may purchase food for consumption from the income they derive from their land. This finding is supported by studies done in west Abaya and Shashemene districts in Southern Ethiopia and in Nepal, which show that cultivated land size influences household food insecurity (14, 18, 19).

The other determinant of household food insecurity in the current study area was the meal frequency of the mothers. Those mothers who had less than three meals were three times more likely to be food insecure than those who had three meals and above. This finding is consistent with the study done in Ataye District, North Shoa zone, Ethiopia (5). The reason might be limited food resources because of household economic problems, so if lactating mothers did not get adequate food during lactation, they were more exposed to undernutrition.

The major strength of this study is highlighting the problems faced by lactating mothers who were responsible for preparing and cooking food for all household members. However, seasonal variation may be one of the limitations of the study since this study was conducted in the postharvest season of Ethiopia. Second, recall bias might have occurred since some of the questions were asked about a past event that might require a good memory. This was minimized by probing the respondents to memorize the event.

## Conclusion

In conclusion, the present study revealed a high level of food insecurity among lactating mothers' households. Residing in rural areas, not having their own source of income, having a meal frequency of less than three, households with poor wealth indices, and having farmland of less than a hectare were factors significantly associated with food insecurity. Thus, the government and other stakeholders need to take action that addresses factors affecting mothers' food security status through strengthening nutrition-sensitive interventions through promoting diversified livelihood development programs like micro-finance or income-generating activities, adoption of agricultural technology, and engaging in non-staple cash initiatives like livestock rearing through strong multi-sectoral collaboration. Further research with strong study designs will also need to come through seasonal variations of household food insecurity among lactating women.

## Data availability statement

The original contributions presented in the study are included in the article/supplementary material, further inquiries can be directed to the corresponding author/s.

## Ethics statement

Ethical approval was obtained from the Institutional Health Research Ethics Review Committee (IHRERC) of the College of Health and Medical Sciences, Haramaya University (Ref.No. 134/2020). Then, the Chiro district health office provided an official letter to local authorities of all selected kebeles. Informed verbal consent was obtained from each participant before the interview. To ensure the confidentiality of participants, anonymous coding was used whereby the name of the participants and any participants' identifier was not written on the questionnaire. Moreover, the rights of members not to take part and not to answer the question they don't want to answer were ensured. Written informed consent to participate in this study was provided by the participants' legal guardian/next of kin.

## Author contributions

Conception and design of the work, acquisition of data, analysis, and interpretation of data were done by SM. Data curation, drafting of the article, revising it critically for intellectual content, validation, and final approval of the version to be published were done by SM, BA, MS, KR, and BT. All authors contributed to the article and approved the submitted version.

## Conflict of interest

The authors declare that the research was conducted in the absence of any commercial or financial relationships that could be construed as a potential conflict of interest.

## Publisher's note

All claims expressed in this article are solely those of the authors and do not necessarily represent those of their affiliated organizations, or those of the publisher, the editors and the reviewers. Any product that may be evaluated in this article, or claim that may be made by its manufacturer, is not guaranteed or endorsed by the publisher.
